# Supplementary material for: Involvement of α-galactosidase OmAGAL2 in planteose hydrolysis during seed germination of Orobanche minor
Source: J Exp Bot. 2021 Dec 1;73(7):1992–2004. doi: 10.1093/jxb/erab527 (PMC8982430; doi:10.1093/jxb/erab527)
Supplement: erab527_suppl_Supplementary_Figures_S1-S6 [file erab527_suppl_supplementary_figures_s1-s6.pdf]

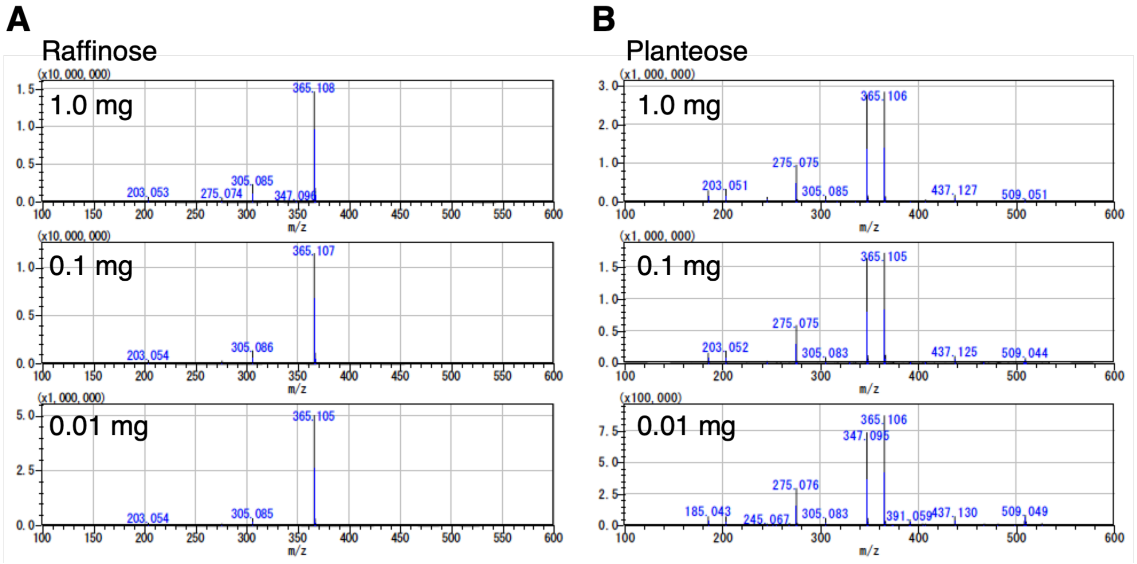

Supplemental Fig. S1. MALDI–MS/MS spectra of raffinose (A), a planteose isomer, and purified planteose (B). 2,5-Dihydroxybenzoic acid solution was mixed with 0.01 mg, 0.1 mg, or 1 mg mL^{-1} of each compound solution and spotted onto ITO glass slides. After crystallization, the spots were analyzed by the iMScope TRIO with *m/z* 527.16 $[M + Na]^+$ as a precursor ion.

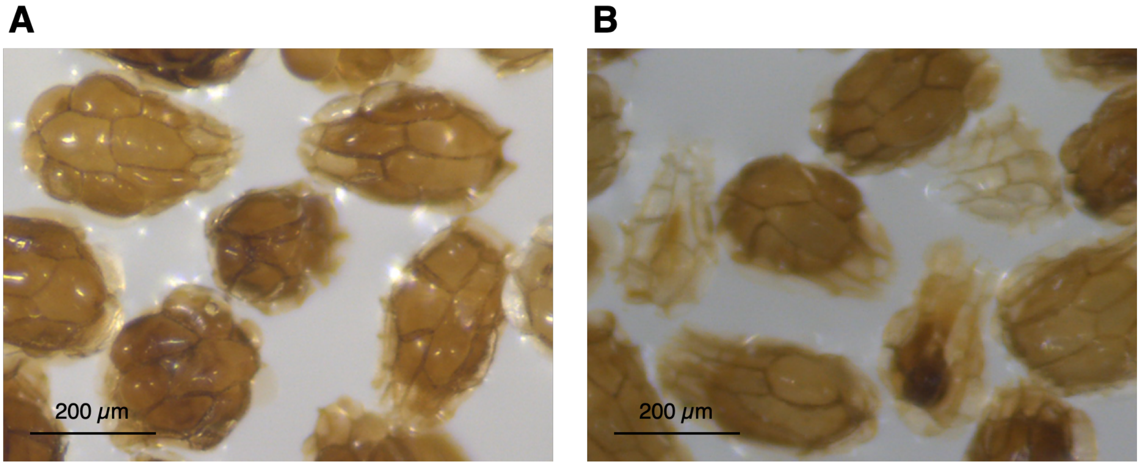

Supplemental Fig. S2. *In vivo* visualization of α-galactosidase activity in germinating seeds of *O. minor* with 5-bromo-4-chloro-3-indolyl-α-d-galactopyranoside (X-α-Gal). The seeds before *rac*-GR24 treatment after conditioning (0 DAT) (A) or 2 DAT (B) were treated with X-α-Gal solution (40 μg mL^{-1}), indicating no pigmentation was observed before radicle emergence.

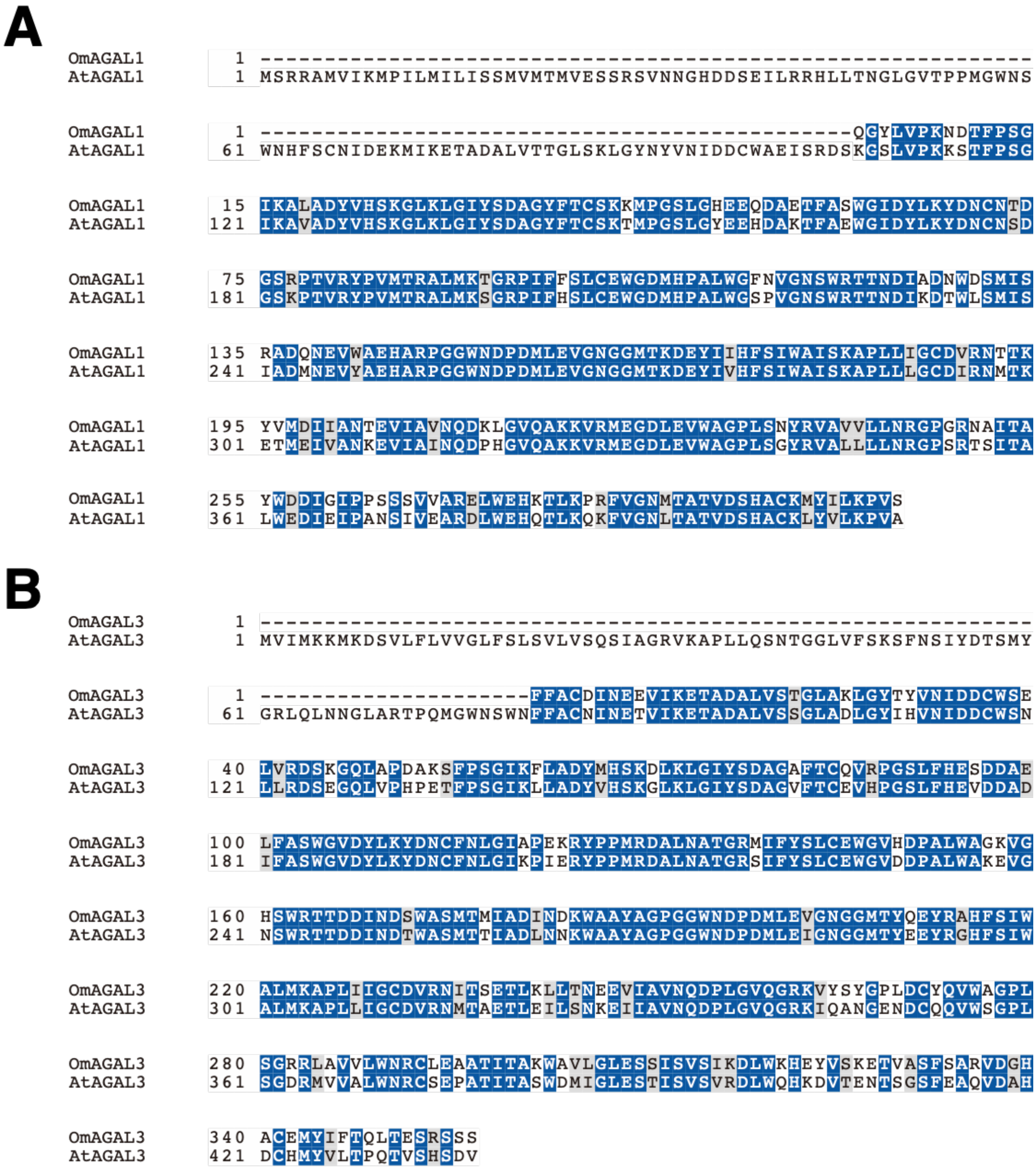

Supplemental Fig. S3. Alignments of a partial sequence of OmAGAL1 (LC636199) with Arabidopsis α-galactosidase AtAGAL1 (At5g08380) (A) and a partial sequence of OmAGAL3 (LC636201) with Arabidopsis α-galactosidase AtAGAL3 (At3g56310) (B) using COVALT (https://www.ncbi.nlm.nih.gov/tools/cobalt/re_cobalt.cgi) and BoxShade (version 3.21, https://embnet.vital-it.ch/software/BOX_form.html). Blue shading indicates identical amino acids, and gray shading indicates similar residues.

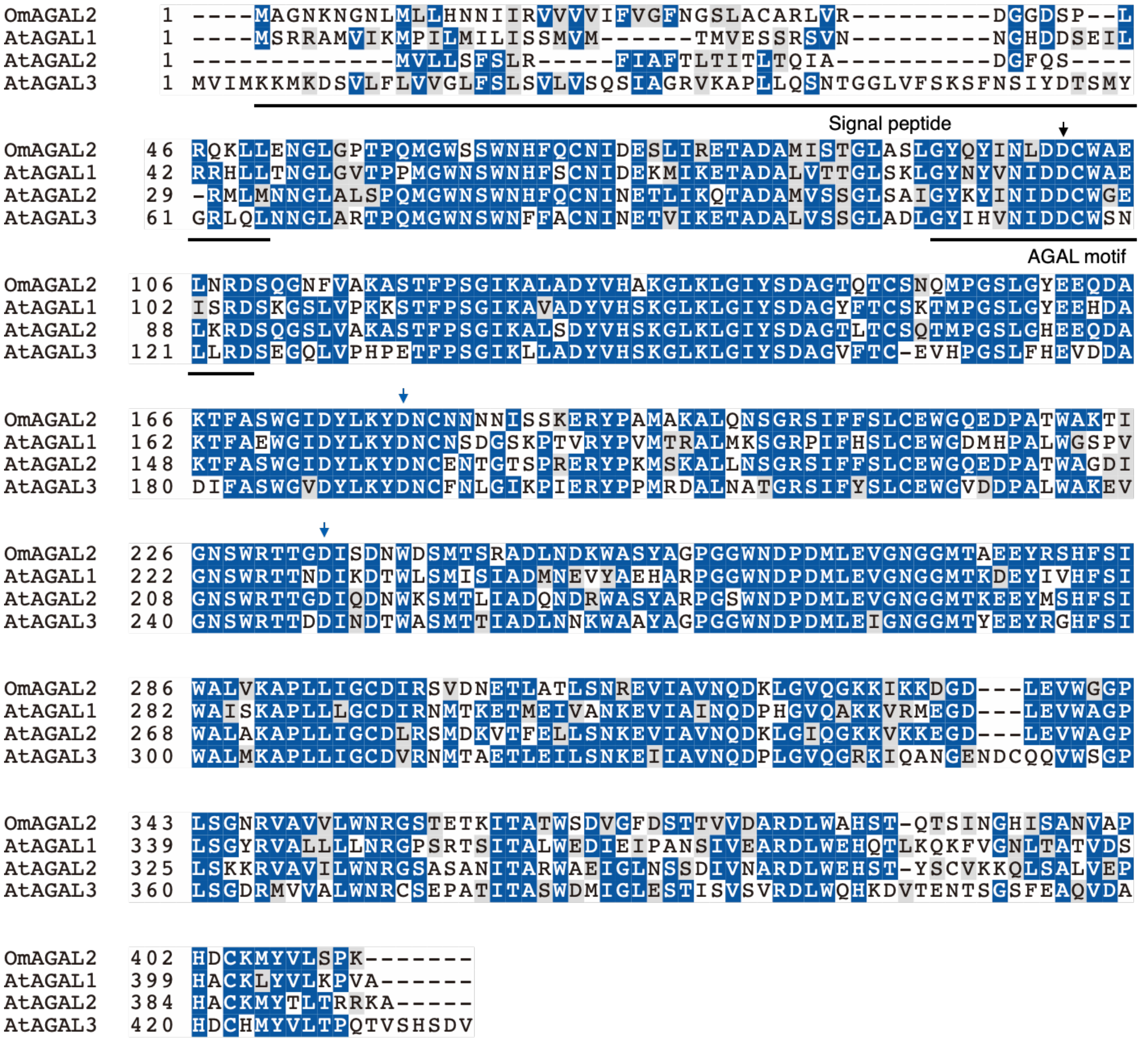

Supplemental Fig. S4. Multiple alignment of OmAGAL2 (LC636200) with three α-galactosidases, AtAGAL1 (At5g08380), AtAGAL2 (At5g08370), and AtAGAL3 (At3g56310), of Arabidopsis using COVALT (https://www.ncbi.nlm.nih.gov/tools/cobalt/re_cobalt.cgi) and BoxShade (version 3.21, https://embnet.vital-it.ch/software/BOX_form.html). Blue shading indicates identical amino acids, and gray shading indicates similar residues. Putative signal peptide and α-galactosidase (AGAL) motif sequences are underlined. A black arrow indicates a conserved aspartate for recognition of galactose in GH27 family enzymes (Imaizumi *et al*, 2017). Blue arrows indicate conserved aspartate residues in active sites (Tapernoux-Lüthi *et al*., 2004; Imaizumi *et al*., 2017).

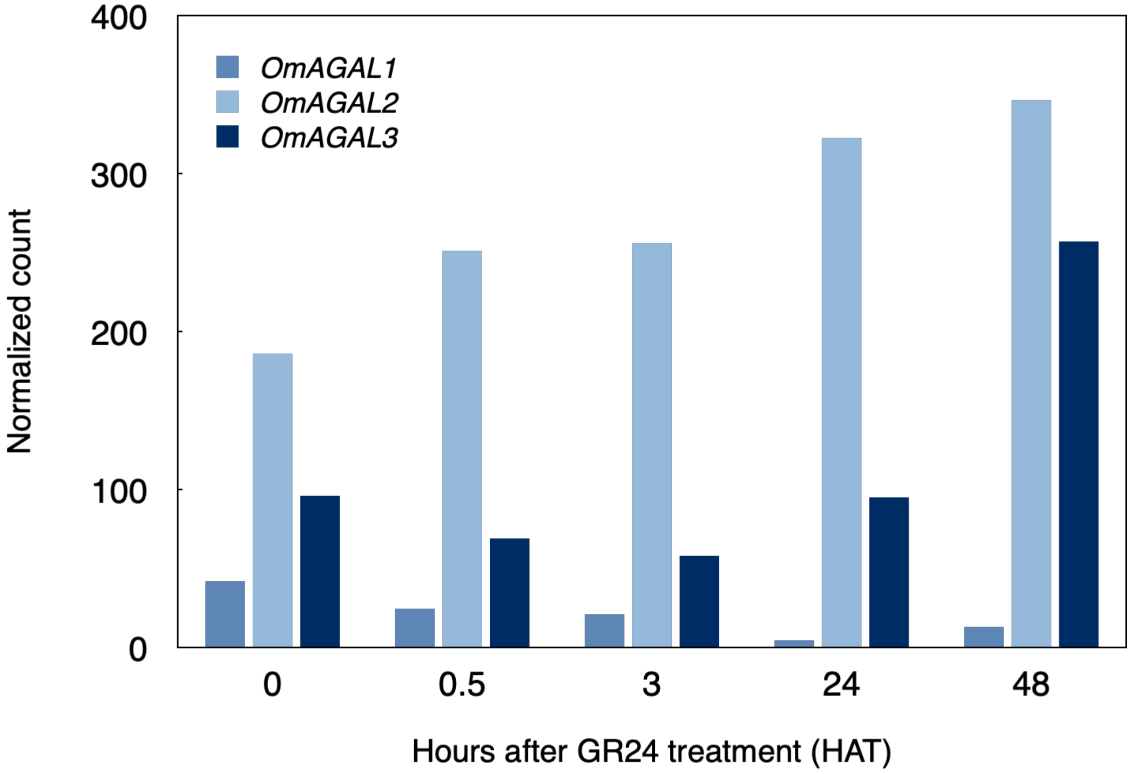

Supplemental Fig. S5. Expression of three α-galactosidase homologous genes in germinating seeds of *Orobanche minor*. Transcriptome analysis using RNA-Seq was conducted for germinating seeds of *O. minor* after conditioning and 0.5–48 h after *rac*-GR24 treatment (Okazawa *et al*., 2020). Three α-galactosidase homologous genes, *OmAGAL1*, *OmAGAL2*, and *OmAGAL3* were identified in the transcriptome.

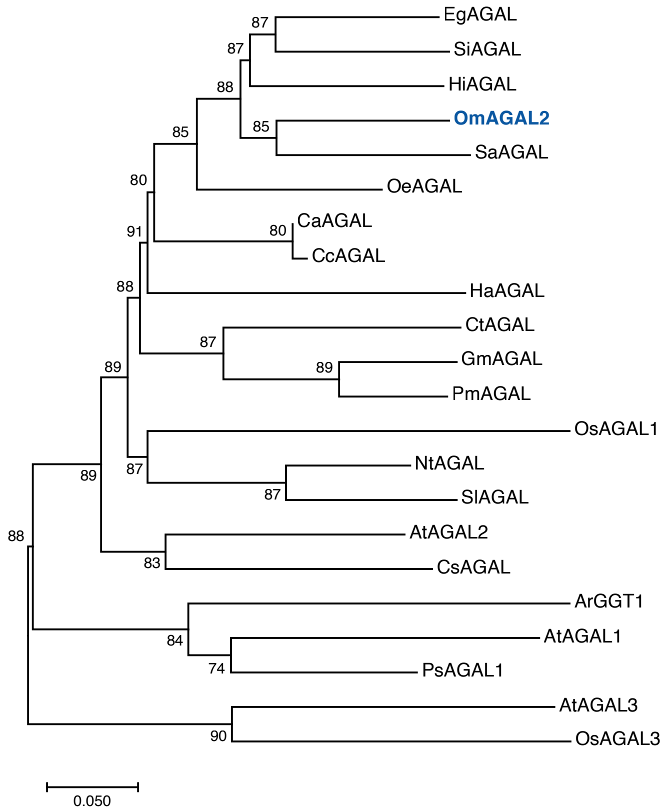

Supplemental Fig. S6. Phylogenetic tree of selected plant α-galactosidases constructed using the neighbor-joining method with MEGAX (version 10.1.8). ArGGT1, *Ajuga reptans* galactan:galactan galactosyltransferae 1 (AAR02007.1); CaAGAL, *Coffea arabica* α-galactosidase (Q42656.1); CcAGAL, *Coffea canephora* α-galactosidase (CAI47560.1); CsAGAL, *Cucumis sativus* α-galactosidase-like precursor (NP_001267530.1); CtAGAL, *Cyamopsis tetragonoloba* α-galactosidase (P14749.1); EgAGAL, *Erythranthe guttata* α-galactosidase (XP_012855605.1); GmAGAL, *Glycine max* α-galactosidase precursor (NP_001235022.1); HaAGAL, *Helianthus annuus* α-galactosidase (BAC66445.1); HiAGAL, *Handroanthus impetiginosus* α-galactosidase (PIN09377.1); NtAGAL, *Nicotiana tabacum* α-galactosidase (AEB98600.1); OeAGAL, *Olea europaea* var. *sylvestris* α-galactosidase-like (XP_022885408.1); OmAGAL2, *Orobanche minor* α-galactosidase 2 (LC636200); OsAGAL1, *Oryza sativa* Japonica Group α-galactosidase isoform X1 (XP_015613565.1); OsAGAL3, *Oryza sativa* Japonica Group α-galactosidase 3 (XP_015646774.1); PsAGAL, *Pisum sativum* α-galactosidase (CAF34023.1); PvAGAL, *Phaseolus vulgaris* α-galactosidase (AAA73964.1); SaAGAL, *Striga asiatica* α-galactosidase (GER38410.1); SiAGAL, *Sesamum indicum* α-galactosidase (XP_011072096.1); and SlAGAL *Solanum lycopersicum* α-galactosidase (AAF04591.1)
